# The feasibility and validity of ambulatory self-report of psychotic symptoms using a smartphone software application

**DOI:** 10.1186/1471-244X-12-172

**Published:** 2012-10-17

**Authors:** Jasper E Palmier-Claus, John Ainsworth, Matthew Machin, Cristine Barrowclough, Graham Dunn, Emma Barkus, Anne Rogers, Til Wykes, Shitij Kapur, Iain Buchan, Emma Salter, Shôn W Lewis

**Affiliations:** 1 School of Community Based Medicine, the University of Manchester, Oxford Road, Manchester, UK; 2 School of Psychology, The University of Manchester, Oxford Road, Manchester, UK; 3 School of Psychology, The University of Wollongong, Oxford Road, Manchester, UK; 4 Institute of Psychiatry, Kings College London, London, UK

**Keywords:** Mobile-phone, Psychosis, Assessment, Ambulant, Schizophrenia

## Abstract

**Background:**

Semi-structured interview scales for psychosis are the gold standard approach to assessing psychotic and other symptoms. However, such assessments have limitations such as recall bias, averaging, insensitivity to change and variable interrater reliability. Ambulant, real-time self-report assessment devices may hold advantages over interview measures, but it needs to be shown that the data thus collected are valid, and the collection method is acceptable, feasible and safe. We report on a monitoring system for the assessment of psychosis using smartphone technology. The primary aims were to: i) assess validity through correlations of item responses with those on widely accepted interview assessments of psychosis, and ii) examine compliance to the procedure in individuals with psychosis of varying severity.

**Methods:**

A total of 44 participants (acute or remitted DSM-4 schizophrenia and related disorders, and prodromal) completed 14 branching self-report items concerning key psychotic symptoms on a touch-screen mobile phone when prompted by an alarm at six pseudo-random times, each day, for one week. Face to face PANSS and CDS interviews were conducted before and after the assessment period blind to the ambulant data.

**Results:**

Compliance as defined by completion of at least 33% of all possible data-points over seven days was 82%. In the 36 compliant participants, 5 items (delusions, hallucinations, suspiciousness, anxiety, hopelessness) showed moderate to strong (*rho* 0.6-0.8) associations with corresponding items from interview rating scales. Four items showed no significant correlation with rating scales: each was an item based on observable behaviour. Ambulant ratings showed excellent test-retest reliability and sensitivity to change.

**Conclusions:**

Ambulatory monitoring of symptoms several times daily using smartphone software applications represents a feasible and valid way of assessing psychotic phenomena for research and clinical management purposes. Further evaluation required over longer assessment periods, in clinical trials and service settings.

## Background

Schizophrenia is distressing and disabling to the individual [1], with an associated cost in the United Kingdom of around 6.7 billion pounds each year [2]. Clinical outcome is usually poor despite treatment, with 80% relapsing by 5 years after the first episode. The major need is for better treatments. Treatment development is slow in this area, with a high rate of failed clinical trials. Currently, we assess treatments by asking patients to recall symptoms over the last 7-28 days, using widely-used semistructured symptom assessments such as the Positive and Negative Syndrome Scale (PANSS [3]) and Calgary Depression Scale (CDS [4]). This introduces bias and averaging, thus clinical information is lost. In addition, standard rating scales require training of raters to ensure high reliability, often difficult to achieve and maintain in multisite studies. For instance, a decrease in between-rater intraclass correlation from 0.9 (“high”) to 0.7 (“acceptable”) on PANSS full-scale score will reduce the power of a study to show an effect from 90% to 72% [5,6]), increasing the risk of a Type 2 error and a failed trial. An advance is needed in the real-time documentation of psychotic symptoms. One under-explored possibility is that of patient self-rating of symptoms.

There is scepticism as to the validity of self-report measures of psychosis. This view is often motivated by knowledge that cognitive deficits [7] and lack of insight [8] are common in patient populations. However, moderate concordance has often been observed between self-report measures and clinician based ratings of psychosis, which has been demonstrated in a range of symptom domains. This includes delusions [9], hallucinations [10], and negative symptoms [11]. Self-report measures may be a more time and cost efficient method of assessing psychosis than clinical interviews, as they do not require the presence of a trained assessor. Thus, self-report measures may be the more attractive option for clinical assessment.

Over the past decade Personal Digital Assistants (PDAs) have been adapted for self-report symptom monitoring in individuals with severe mental illness [12]. Studies evaluating PDAs have shown low rates of drop-out in community dwelling individuals with psychotic disorders [13-15]. For example, Granholm and colleagues [13] found that 87% of patients were compliant to PDA based momentary assessment as defined as completing at least four out of 28 data-points. Other studies have observed similarly low rates of drop-out when using more conservative definitions of compliance (eight out of 28 data-points) [15].

PDAs are offline systems and whilst the data is collected in the real world it cannot be assessed until brought into the laboratory/clinic and downloaded. Assessing data in *vivo* is desirable in that it could help to facilitate earlier and more immediate intervention, which in turn could help to reduce relapse, self-injury and the need for unscheduled acute care. Automated and personalised feedback could help clinicians to devise and review treatment strategies prior to consultation allowing for more effective care. An appropriately enabled mobile phone may have the advantage that people are accustomed to carrying and recharging it and are often familiar with the technology. Software applications are also easily uploaded to participants’ own smartphones ensuring that the individual does not have to carry with them an additional device. In a recent Ofcom report in the United Kingdom, 27% of adults and 47% of teenagers currently owned a smartphone [16]. With advances in mobile phone technology PDAs are becoming increasingly obsolescent.

The first objective of this study was to evaluate and validate new mobile phone based self-report assessment scales for psychosis against the PANSS and the CDS, both widely used retrospective interview assessments of psychotic and related symptoms and considered to be benchmark scales accepted by regulatory authorities in clinical trials. Scales were specifically developed for purpose-built smartphone assessment software (i.e. ClinTouch) in order to monitor psychotic symptoms in real time. The second objective was to assess feasibility and acceptability to patients with serious mental illness, examining levels of compliance and drop-out to the procedure in individuals at different stages of psychosis. We also aimed to examine the internal consistency of the scales and their instability over time. In order to gauge the feasibility of installing this software onto participants own phones, we assessed the extent to which participants used mobile-phone technology in their everyday lives. In order to assess safety, in that that this approach did not cause distress, we assessed “reactivity” to the method as reported by participants at the end of sampling.

Thus, the study had two main hypotheses. First, that symptom data collected over a smartphone software application would show good correlations with corresponding data collected by conventional, gold standard rating scales. Second, high levels of compliance and low dropout from smartphone based assessment would be possible in individuals at different stages of psychosis (ultra-high risk, acute and remitted). In this study we also predicted that the self-report scales would show high internal consistency (*α* coefficients), but be sensitive to change, as represented by instability across time-points. No predictions were made as to participant’s level of phone use in their everyday lives.

## Method

### Participants

In order to fully assess usability and the validity of collected data, we chose three clinical subgroups of patients who represent different severities and stages of the disorder and are commonly the focus for clinical trials. Group one consisted of patients meeting the criteria for a Diagnostics and Statistical Manual (Fourth Edition; DSM-IV) diagnosis of schizophrenia, schizoaffective disorder, delusional disorder or schizophreniform disorder and were in partial or full remission, as defined by having mild or absent positive symptoms and being on stable antipsychotic medication for at least three months. Group two consisted of acutely psychotic patients with the same diagnoses, but who were within four weeks of starting, restarting or changing their medication because of worsened symptoms or within four weeks of a hospital admission. Group three comprised of individuals who had met criteria for being at ultra-high risk of developing psychosis at some point during the past year (“prodromal”) according to the Comprehensive Assessment of At Risk Mental State [17,18] and who were not currently on antipsychotic medication: 50% of these participants still met the CAARMS criteria for the ultra-high risk (UHR) mental state at the time of taking part in the study. Organic and substance induced psychosis were exclusion criteria for all three groups. Full demographic and clinical information is provided in Table 1. Eligible participants were prospectively recruited into the three groups until each group contained 12 subjects who had managed to complete at least 33% of the 42 data entry points possible during the six consecutive days of testing in accordance with momentary assessment studies [19].

**Table 1:** Demographic and clinical information for sample

	**Acute (*n*=12)**	**Remitted (*n*=12)**	**UHR (*n*=12)**
Age, mean (*SD*)	36.8 (10.0)	35.5 (8.0)	22.0 (4.4)
Males, *n*	9	9	10
History of CBT, *n*	5	7	3
Acute admissions, mean *(SD)*	4.8 (4.8)	2.2 (1.7)	0
Age at first contact with clinical			
services, mean *(SD)*	25.9 (8.2)	27.2 (6.8)	18.0 (6.2)
Years of education, mean (*SD*)	11.7 (2.5)	12.0 (3.0)	12.6 (1.6)
Ethnicity, *n*			
White British	8	11	10
Black British	4	0	0
Asian Pakistani	0	1	1
Middle Eastern	0	0	1
Diagnosis, *n*			
Schizophrenia	11	8	0
Schizoaffective	1	2	0
Schizophreniform	0	2	0
Medication, *n*			
Atypical AP	9	8	0
Typical AP	0	1	0
Typical & atypical AP	3	3	0
Antidepressant	7	6	4
Living status, *n*			
Alone	0	6	2
Ward	10	0	0
Family	2	3	7
Partner	0	1	3
Shared living	0	1	0
Supported living	0	1	0
Service recruited through, *n*			
CMHT*	0	7	0
Early intervention	2	3	10
Inpatient ward	10	0	0
Early detection	0	0	2
Assertive outreach	0	1	0
Rehabilitation services	0	1	0
Interview total scores, mean (*SD*)			
PANSS	63.4 (13.0)	49.9 (9.9)	56.8 (13.6)
CDS	14.0 (5.2)	12.1 (2.8)	13.9 (4.7)

*Community Mental health team.

### Equipment

The assessment software was developed specifically for touch screen Android mobile phones. Android is an open source operating system developed by Google that runs on a range of phones from different manufacturers such as HTC, Samsung and Sony Ericsson. Android devices are becoming increasingly widespread in the mobile phone market and it is expected to be the most popular mobile operating system by the end of 2011 (http://www.gartner.com/it/page.jsp?id=1622614). For this trial we chose to use the Orange San Francisco device, although the software was developed to work with any compatible Android based phone.

#### Measures

##### Semi-structured interviews

The “gold standard” Positive and Negative Syndrome Scale (PANSS) and Calgary Depression Scale (CDS) assessments were administered to each subject at baseline, and again after completion of the six day data collection period. The PANSS (Kay et al., 1987) is a semi-structured interview where a range of positive (7 items), negative (7 items) and general symptoms (16 items) are rated on a seven point scale (1 = absence; 7 = severe). Its validity and reliability have been demonstrated (Bell et al., 1992; Kay et al., 1987). An experienced researcher (JPC) was blind to mobile assessment scores during the debriefing interview (unblinded once). Excellent inter-rater reliability was demonstrated with an independent rater rating 12 (5 acute, 3 remitted and 4 UHR) of the audio-recorded PANSS interviews (Spearman’s correlations, PANSS positive subscale, *rho* = .91; negative, *rho* = .82; global, *rho* = .81; total, *rho* = .79).

The CDS assesses depression and related manifestations, and contains 9 items scored from absent (1) to severe (4). It has good internal consistency and convergent validity with other measures of depression, and effectively discriminates the presence or absence of co-morbid depression [4,20]. In this study the CDS was used to assess the previous week in order to cover the 7 day time sampling procedure.

##### Mobile phone assessment questions

**Figure 1:**
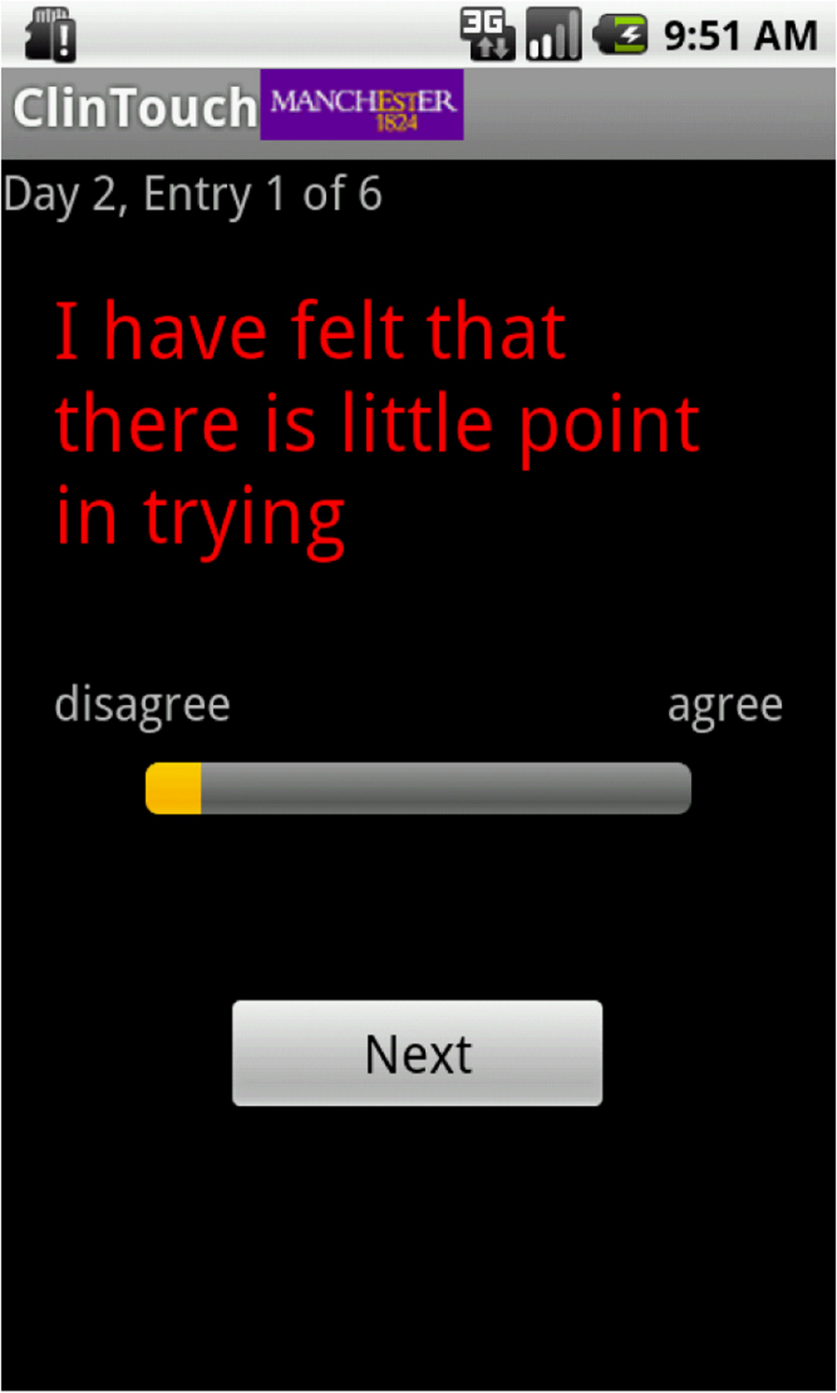
A screenshot of the question response page.

The mobile phone assessment items were designed to be equivalent to 12 items of the PANSS and 2 items of the CDS, and are displayed in Additional file 1. The items were selected to give an appropriate range of key positive and mood symptoms. Participants were required to respond on an analogue scale indicating the degree to which they agreed or disagreed with statements relating to their symptoms since the last entry (see Figure 1). This was, therefore, not an experience sampling study, where questions would have related to the present moment in time. Although these reports were retrospective, the time between an event and its recollection was minimal, reducing the effects of memory bias. Retrospective ambulant assessment may better capture infrequent, but nevertheless important, clinical phenomena that would otherwise be missed at the time of entry. For example, assessments at the time of entry may miss perceptual abnormalities in individuals at UHR of psychosis (unpublished observation). The first entry of the day related to the period of time since wakening.

In order to reduce the length of time taken to complete the items, these were divided into two sets, displayed at alternative time-points. Guilt, hopelessness, depression, social withdrawal, conceptual disorganisation, excitement and hallucinations were assessed in set one, whereas anxiety, grandiosity, hostility, somatic concern, guilty ideas of reference, paranoia and delusions were assessed in set two. The allocation of scales to the two sets was based on the need to assess overlapping symptom domains (e.g. paranoia and delusions) at the same time-point and to keep the number of items balanced. Some of the self-report scales were branched so that the use of certain items was contingent on the participant’s previous response. The stem and branching questions related to the constructs measured on the PANSS and the CDS items, while being compatible with self-report. Thus, 15 to 30 questions were presented in set one and 11 to 31 questions were presented in set two.

When developing frequently repeated symptom scales it is necessary to keep the number of items to a minimum in order to reduce burden on participants [21]. However, a wide range of delusional beliefs have been reported in patient populations [22] making this problematic. Therefore, ClinTouch was equipped with a ‘delusion’ menu on the admin page, which allowed the researcher to personalise which delusions a participant was currently experiencing based on the initial PANSS interview and reports by clinical staff. The selected delusion then populated the questions that were administered, which were scored for preoccupation, distress and behavioural impact (see Additional file 1). A maximum of two delusions could be entered for each participant. For participants who were experiencing three or more delusions those two associated with the greatest conviction and distress were entered into the ClinTouch software. The frequency of different delusions were: ‘I have felt like other people could read my thoughts’ (*n*=6), ‘I have felt like my thoughts were being controlled or influenced’ (*n*=6), ‘I have felt like I could read other people’s thoughts’ (*n*=4), ‘I have felt like people were not what they seemed’ (*n*=2), ‘I have felt like things on the TV, radio or magazines had a special meaning for me’ (*n*=2) and ‘I have felt like there was a conspiracy against me’ (*n*=2).

Two items were included on the mobile phone to assess safety, or “reactivity” to the methodology [23]. These items were ‘keeping the diary has influenced my thoughts’ and ‘keeping the diary has influenced my mood’. Important to note is that these questions did not measure the direction of the reactivity (i.e. whether it made someone feel better or worse).

#### Procedure

**Figure 2:**
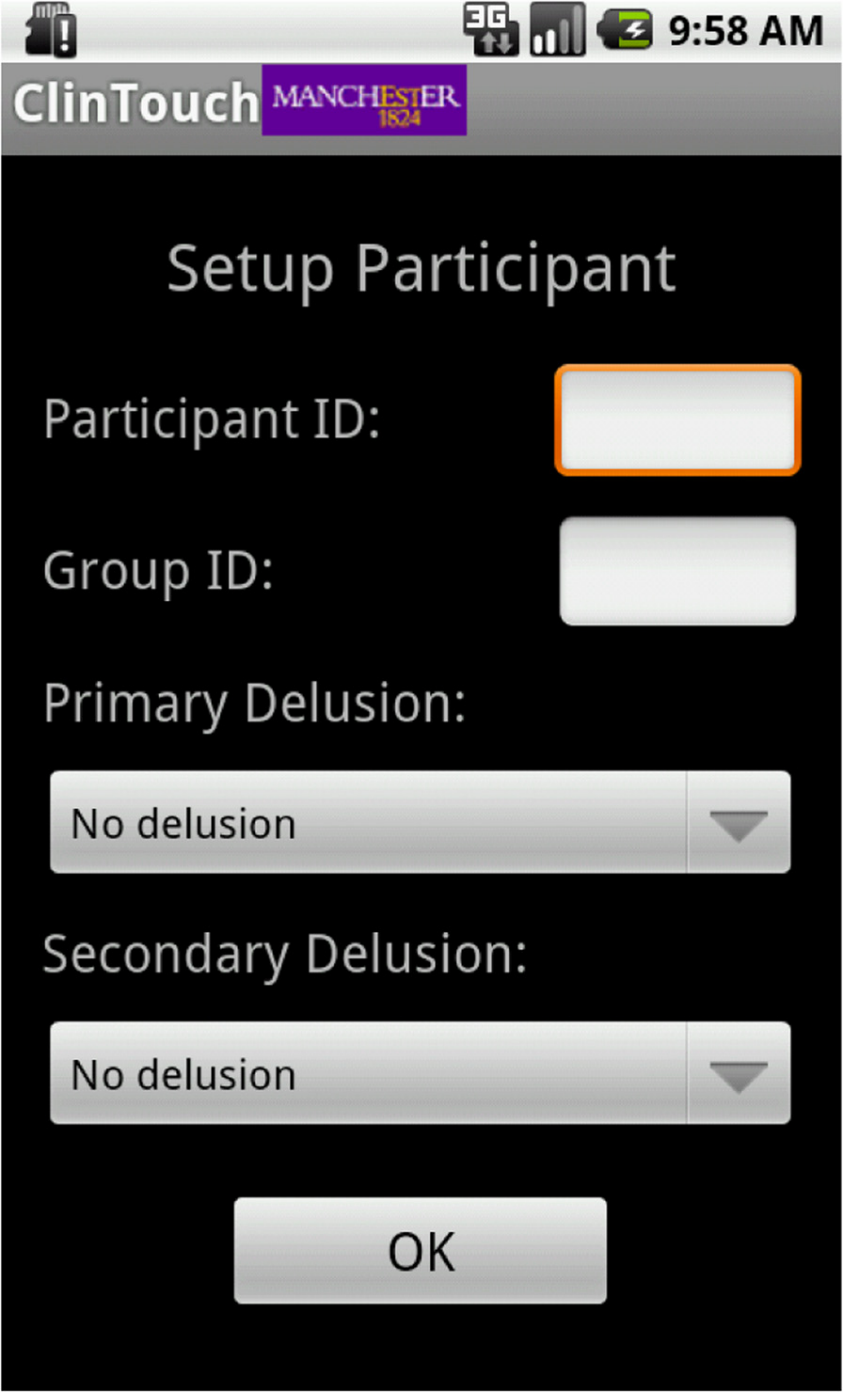
A screenshot of the researcher administration page.

The study received approval from the North West One National Health Service Research Ethics Committee (ref: 11/H1017/3). The purpose of the briefing session was to obtain written consent, complete the initial PANSS and CDS interviews, and run training in the ClinTouch software (including practise questions). At the end of this session, participant number, group identity (acute, remitted and UHR), alarm volume, and delusion type were also entered onto the device via a password protected admin screen (Figure 2).

The assessment procedure started on the morning following the briefing session. The ClinTouch software caused the mobile phone handsets to emit an alarm and vibrate at six pseudo-random times of the day (generated by a random number generator stratified within set epochs of time at least one hour apart) between 9:00 and 21:00 hours. It also triggered a ‘start questions’ icon on the touch screen. The participant was given the option to trigger a repeat alarm 5 minutes later if they were occupied (a ‘snooze function’). All participants had 15 minutes from the initial alarm within which to complete the questions. A pseudo-random, as opposed to fixed, sampling schedule was thought to be advantageous since it facilitated the assessment of a wide range of situations and times of the day and prevented individuals from greatly changing their activities in order to account for completing the questions [23]. Forced entry times were also expected to reduce response bias (e.g. only completing the diary when asymptomatic). The researcher telephoned participants once or twice during the week (participant’s preference) to gauge acceptability, offer encouragement, and remind them to charge the device. Participants were able to access all applications on the mobile phone devices (e.g. games, camera), but at this proof-of-concept stage of the study the devices had no wireless connectivity. Important to note is that the lack of connectivity made no difference to the way that the software operated from a user perspective.

Upon completion of the 7 day momentary assessment procedure the researcher met with the participant to re-administer the PANSS and CDS, and to assess general mobile phone usage outside of the sampling procedure. UHR individuals were also assessed with the CAARMS in order to ascertain whether they currently met this criteria.

#### Statistics

All analysis was performed in Stata 10.0 [24] and SPSS 15.0 [25]. First, the analogue scales for each mobile-phone assessment item were converted to 7-point Likert scales by the computer software for the purpose of the analysis. Grandiosity item 1 (‘Compared to the average person, I am: (analogue scale: worse - better)’) was transformed so that only positive (grandiose) appraisals of oneself contributed to the gradient of the score (i.e. 1-4 coded as 1, 5 coded as 2, 6 coded as 3, 7 coded as 4).

In order to assess the multifaceted nature of the constructs measured by each PANSS scale, there were multiple ambulant assessment items. For example, the Anxiety momentary assessment scale consisted of four items. The mean of these items was then calculated to constitute the individual’s momentary assessment score for that symptom domain or scale (e.g. the mean of items 1, 2, 3 and 4) at a particular time-point. In some cases, in order to better correlate with the PANSS, symptom scales were supplemented by ratings on other scales. The delusion mean score was calculated from the delusion items, and the mean score of the grandiosity, somatic concern and suspiciousness scales (e.g. item 1 + item 2… + grandiosity^mean^ + somatic concern^mean^ + suspiciousness^mean^). Reports of grandiosity, somatic concern and suspiciousness all inform the rating of the delusion item on the PANSS interview. The depression scale comprised of the depression items and the mean hopelessness score (i.e. item 1 + item 2… + hopelessness^mean^). Feelings of hopelessness are part of the criteria for scoring a 4 or greater on the PANSS depression scale (item g6).

All analyses were performed at a person-level. Therefore, a mean symptom score was calculated for each individual from across all available data-points, resulting in a single score for each participant. Logistic and regression models (Enter method) were carried out to establish interview and demographic predictors of drop-out (completion of ≥33% of all available data-points) and diary entries completed respectively. As the person level mean symptom scores were positively skewed, non-parametric Spearman’s correlations established the degree to which mean diary scores resembled their corresponding interview subscales. Cronbach’s α was used to calculate the reliability across items for each scale. In order to measure the instability of the different constructs, the mean squared successive difference (*MSSD)* and standard deviation (*SD)* were calculated from across all available data-points for each individual [26], including data point across days and those which were not sequential due to missing time-points. The *MSSD* and *SD* have been recommended as valid metrics of instability, which are widely used in clinical research [27].

## Results

### Adherence to the methodology

Initial verbal approach to participate was made by a member of the clinical care team and about 50% of those approached declined to take part. Of the 51 patients who agreed to be contacted about the study and had their contact details passed on to the research team, four subsequently declined, two were ineligible and one could no longer be contacted.

Compliance to the methodology was defined as completing at least 33% (14 or more) of all possible (42) entries. In all, 44 participants consented to and entered the study to ensure that 36 met this compliance criterion after 7 days: in other words, 82% of participants met the compliance criterion. Six acute and two remitted patients with psychosis failed to meet this criterion (Mean age: 31.5 (*SD*; 11.1), all male). Logistic regression analysis was performed to examine whether positive, negative and general subscales on the PANSS (prior to sampling), CDS total score (prior to sampling), or age significantly predicted whether an individual was compliant with the methodology. Positive symptom subscale severity was the only significant predictor (*OR* = 0.68, *p* = .033, *CI*: 0.48 – 0.97). The 8 non-compliant participants are excluded from all analyses subsequently presented in this manuscript.

A high number of entries were completed by the 12 acute (Min = 14, Max = 41, Mean = 28.5, *SD* = 8.1), 12 remitted (Min = 14, Max = 40, Mean = 29.5, *SD* = 9.3) and 12 UHR (Min = 21, Max = 38, Mean = 31.1, *SD* = 6.6) participants who were compliant with the procedure. Thus, on average, the aggregated sample completed 31.1 of all possible data-points (72%). A one-way ANOVA showed these differences to be non-significant across groups (*F* (2,35) = .312, *p* = .734). Multiple regression analysis was performed to investigate whether age, gender, PANSS subscales, and CDS total predicted the total number of diary entries completed by each individual. There were no statistically significant predictors.

### Reactivity to the method

Reactivity (changes in thoughts or emotions) to filling in the questions was greatest in the acute group (mean: 3.6 (*SD*: 2.4)), and greater in the remitted (mean: 2.9 (*SD*: 1.5)) compared to UHR individuals (mean: 2.4 (*SD*: 1.7)). A Kruskall-Wallis test showed this difference to be statistically non-significant (x^2^ = 3.351 (df: 2), *p* = .187). Regression analysis was used to assess whether positive, negative or general symptoms on the PANSS, or CDS total score, significantly predicted reactivity across all three groups. Only negative symptoms predicted greater reactivity to the method (*β* =.54, *p* = .001).

### Correlation between momentary assessment and interview subscales

Summary statistics for the mobile-phone assessment items and clinical interviews are provided in Table 2. The standard deviation *(SD)* score reported in this table represents variability between individuals’ mean scores (not within individual variability). The CDS item 2, guilty ideas of reference, was only ever endorsed by two participants and was therefore not analysed.

**Table 2:** Summary statistics for interview and diary subscales, and the results to Spearman's correlations (in order of strength)

	**Chronbach's alpha**	**Diary scores**				**Interview scores**				**Correlation between interview and diary scores**	
**Questions:**		**Min**	**Max**	**Mean**	***SD***	**Min**	**Max**	**Mean**	***SD***	***rho***	***p*-value**
Hopelessness (*CDS*)	0.87	1.0	6.7	3.3	1.5	1.0	3.0	1.8	0.8	0.80	*p* <.001
Delusions	0.93	1.0	5.3	2.0	1.3	1.0	5.0	2.6	1.3	0.74	*p* <.001
Anxiety	0.96	1.0	6.0	2.7	1.5	1.0	5.0	3.0	1.1	0.69	*p* < .001
Hallucinations	0.96	1.0	6.4	2.6	1.7	1.0	5.0	2.4	1.5	0.68	*p* < .001
Suspiciousness	0.95	1.0	6.2	1.0	6.2	1.0	5.0	2.5	1.3	0.63	*p* <.001
Grandiosity	0.76	1.0	4.2	2.0	1.1	1.0	4.0	1.5	1.0	0.53	*p* <.001
Depression	0.83	1.1	5.9	3.4	1.2	1.0	4.0	3.0	1.2	0.45*	0.006*
Guilt	0.95	1.0	5.7	2.0	1.2	1.0	5.0	1.7	1.1	0.44	0.006
Somatic concern	0.96	1.0	7.0	3.1	2.1	1.0	5.0	1.7	1.1	0.39	0.019
Passive apathetic social withdrawal	0.93	1.0	6.9	4.1	1.6	1.0	3.0	1.8	0.9	0.26	0.131
Hostility	0.86	1.0	5.0	2.4	1.2	1.0	7.0	1.9	1.2	0.25	0.145
Excitement	0.89	1.0	6.8	3.7	1.7	1.0	4.0	1.5	0.9	0.06	0.712
Conceptual disorganisation	0.95	1.0	5.0	2.0	1.1	1.0	3.0	1.6	0.8	-0.04	0.832

*Represents correlation with item G6 on the PANSS. Correlation with item 1 on the CDS (mean = 2, SD = .7, Min = 1, Max = 4) was *rho* = .50, *p* = .002

Min = minimum, Max = maximum, *SD* = standard deviation, PANSS = Positive and Negative Syndrome Scale, CDS = Calgary Depression Scale.

The strength of the associations between the diary and corresponding interview subscales varied considerably (Table 2). Hopelessness, delusions, anxiety, hallucinations and suspiciousness diary items showed strong Spearman’s correlations with the corresponding items on the CDS and the PANSS (*rho* > .60). Moderate and still statistically significant correlations were also observed for grandiosity, depression, guilt, and somatic concern (*rho* > .35). However, passive and apathetic social withdrawal, hostility, excitement, and cognitive disorganisation were not significantly correlated with their corresponding PANSS subscales.

### The internal consistency and instability of the scales

As can be seen in Table 2, the alpha scores for each of momentary assessment scales were high suggesting good internal consistency. The *MSSD* and *SD* scores for each momentary assessment scale are displayed in Table 3. A greater score represents greater instability across time. The delusion instability score was only calculated in individuals who triggered the delusion questions at briefing. All momentary assessment scales showed some instability across time. Passive apathetic social withdrawal was the least stable subscale, followed by excitement, conceptual disorganisation and anxiety. Delusions and grandiosity were the most stable self-report scales.

**Table 3:** With in subject instability metrics for all scales

**Questions:**	***MSSD (SD)***	***SD (SD)***
Hopelessness - CDS	1.3 (1.4)	0.8 (0.5)
Delusions	0.7 (1.3)	0.5 (0.4)
Anxiety	1.9 (1.7)	1.1 (0.6)
Hallucinations	1.2 (1.6)	0.6 (0.5)
Suspiciousness	1.1 (1.9)	0.6 (0.5)
Grandiosity	0.9 (1.1)	0.6 (0.5)
Depression	1.0 (1.0)	0.8 (0.4)
Guilt	1.8 (2.1)	0.9 (0.6)
Somatic concern	1.2 (1.4)	0.7 (0.6)
Passive apathetic social withdrawal	2.7 (2.0)	1.3 (0.6)
Hostility	1.9 (2.7)	0.9 (0.5)
Excitement	2.2 (2.7)	1.0 (0.6)
Conceptual disorganisation	2.1 (2.3)	0.9 (0.6)

*MSSD*: mean squared successive difference.

*SD*: Standard Deviation.

*CDS*: Calgary Depression Scale.

Note: *SD* in brackets represents variability between participants on these scores.

### General phone usage of the sample

A series of questions were asked to gauge the feasibility of using the ClinTouch software on participants’ own phones. Of the sample of 36, 83.3% currently owned a mobile phone, and 44.4% owned a smart phone (30.6% with a touch screen). Phone use included individuals who were acute (66.7%), remitted (91.7%) and UHR (91.7%). 63.9% of individuals with mobile phones reported that they kept these on them all or most of the time, with an identical number usually or always taking their phones with them when they went out. The sample had owned a mean of 8.3 (*SD* = 7.0) mobile-phones devices. 86.1% of the current sample reported that they would buy a new phone in the future.

## Discussion

This study attempted to examine the validity and feasibility of a self-report scale for assessing psychotic symptoms on appropriately enabled mobile phones. The results suggest that the methodology is both feasible and acceptable across different stages of psychosis. Additionally, the data support the validity and reliability of several of the momentary items, suggesting that they pose a useful alternative to traditional symptom assessment.

The number of individuals dropping out of the study was relatively low across remitted and UHR samples, although slightly elevated in acute patients, where a third of individuals were non-compliant. This may explain the finding that positive symptoms significantly predicted non-compliance to the procedure. This supports the notion that momentary assessment is a relatively demanding approach, to which certain more symptomatic and chaotic patients may have difficulty in remaining compliant [21]. Thus, in acute settings it may be beneficial to adapt the momentary assessment procedure (e.g. sampling rate, item number) to individual’s preferences and needs, or use an alternative method of assessment.

In compliant individuals, the number of assessment occasions was relatively high and similar to past momentary assessment research using PDAs in this population. For example, Swendsen and colleagues [15] observed an identical completion rate of 72% of all data-point completed, whereas Granholm and colleagues [13] found this to be 69%. In our study the number of entries was non-significantly different between the groups, suggesting that although a subgroup of acute patients struggled to complete the minimum number of entries, the majority were just as able to comply with the procedure as those with more attenuated symptoms. It should be noted that although compliance was high in this study rates of refusal to initially take part could not be assessed. Furthermore, socioeconomic status and reading ability were not considered, which may have predicted levels of non-compliance.

Reactivity to the methodology was minimal across the groups, although it was slightly elevated in individuals with greater levels of negative symptoms. This may explain why these symptoms have been found to predict drop-out in experience sampling studies (unpublished observation). Important to note is that reactivity could not be assessed in individuals who dropped out of this study and did not complete any diary entries. It is possible that greater levels of reactivity may be observed in non-compliant participants.

In line with the hypotheses, correlations with PANSS and CDS subscales were mainly significant, although there was considerable variability. Positive symptom scales (i.e. delusions, hallucinations, grandiosity, somatic concern and suspiciousness) generally showed moderate to strong correlations with their corresponding PANSS scales. Affective symptoms, including hopelessness, anxiety, guilt and depression, also significantly correlated with the interview measures. Therefore, ClinTouch appears to collect data which is comparable to traditionally used, gold standard assessments of psychotic symptoms and mood.

Passive apathetic social withdrawal, excitement, hostility and cognitive disorganisation items showed weak and non-significant correlations with their corresponding interview scores, requiring further consideration. There are several possible reasons for this finding. Most important is that the equivalent PANSS item ratings are based largely on observable behaviour during the interview, often supplemented by the reports of clinical staff and family members. Replicating this in a self-report item is a challenge. This is not to say that either holds a more valid or clinically useful viewpoint, but rather that they assess different constructs. Also, hostility and excitement represent socially undesirable behaviours, which patients may not associate with themselves or may wish to underplay in self-report measures. Finally, there was a limited range of scores observed on the apathetic social withdrawal and cognitive disorganisation PANSS subscales, which may have attenuated the correlations with the momentary assessment scales.

All of the mobile phone self-report scales showed instability (ie fluctuations) across time as shown by high within subject *MSSD* and *SD* scores, suggesting that they were sensitive to subtle shifts in symptomatology. Indeed, the mood scales (i.e. anxiety, depression and guilt) showed equivalent or greater levels of instability than typically employed experience sampling scales [27]. Delusions and grandiosity were the most stable across time potentially suggesting that these reflect relatively fixed and inflexible belief systems. Passive apathetic social withdrawal showed the greatest instability, perhaps representing changes in the individual’s inclination to be around others. All of the self-report scales also showed good internal consistency.

The advantages of using technology to monitor mental illness have recently been documented [28,29]. Ambulant monitoring provides detailed information about an individual’s symptoms across a variety of situations and times of the day. This could generate discussion points for consultation; identify ‘relapse signatures’; and highlight momentary symptom triggers. It could also be used to monitor real-time acute phase medication treatment effects in the early stages of intervention [30]. This is important given that most clinical improvement is now known to occur within the first 7-days after receiving antipsychotic treatment [31,32]. Furthermore, mobile assessment techniques can be adapted for use alongside psychosocial intervention [33]. For example, person-tailored interventions could be triggered when an individual’s symptom score reaches a certain threshold or to facilitate ‘homework’ [34]. In research, it will also potentially allow better clinical phenotyping, and stratification for clinical trials.

Perhaps the greatest strengths of ClinTouch are that it offers automatic wireless uploading of clinical information to a central server and can be installed on patients’ own phones, thus obviating the need to carry a special purpose device. Furthermore, smartphone technology may be more user-friendly and time-efficient than text-based systems [35]. We observed that the majority of this sample currently owned and regularly used mobile phone technology, many of which were smart phones. With advances in technology it is likely that advanced mobile phones will become increasingly affordable and widespread, and this will make it a viable option for clinical assessment within clinical services. Future research will need to evaluate the merits and pitfalls of this approach.

Previous research in the area of telehealth and telecare devises suggests a need for deeper understanding of how ClinTouch is used in practice to identify the factors that facilitate implementation of this device. As the field of new technology in mental health aspires to moves beyond demonstration and towards the embedding of devices such as ClinTouch in everyday clinical practice, there is a need to engage methods and sub-studies that are able to describe the processes, identifying facilitators to context specific and successful implementation of telecare [36]. Qualitative methods are being used to consider the social practices behind the integration and incorporation of the ClinTouch technology. Understanding their interactions with professionals and the synergy or otherwise with clinical expectations will inform its future use.

## Conclusions

ClinTouch is a valid form of self-assessment, which could facilitate the real-time monitoring of symptoms in schizophrenia in research and clinical management settings. In addition to overcoming the constraints of rater training and limited reliability, recall bias and averaging, it potentially offers advantages over semi-structured interview administered scales allowing finer-grained analysis over briefer time periods, with potential inclusion of external contingency data, diurnal and short-term variability and adding in of other behavioural data gathered by the same device, such as sleep pattern and activity. Limitations currently include restricted ability to assess negative and behavioural symptoms. Further pilot testing is required to assess whether it can be used to monitor symptoms over a longer period of time or treatment effects.

## Competing interests

No competing interests, financial or otherwise, arise from this research.

## Author’s contributions

JPC recruited and assessed all participants, analysed the data and co-wrote the manuscript. JA was project manager and principal engineer overseeing software design, development and deployment and helped prepare this manuscript. MM created and designed the mobile-phone software, and assisted with the writing up of the findings. CB, GD, AR, TW, IB, and SK sat on the steering committee for this project, assisting with its design and commenting on several drafts of this manuscript. ES aided with the writing of the manuscript and reviewed the literature on self-report assessments of psychosis. SL was the primary investigator and grant holder. He designed the study and co-wrote this manuscript. All authors read and approved the final manuscript.

## Pre-publication history

The pre-publication history for this paper can be accessed here:

http://www.biomedcentral.com/1471-244X/12/172/prepub

## Supplementary Material

Additional file 1 Momentary assessment items. Click here for file
