# Supplementary material for: The feasibility and validity of ambulatory self-report of psychotic symptoms using a smartphone software application
Source: BMC Psychiatry. 2012 Oct 17;12:172. doi: 10.1186/1471-244X-12-172 (PMC3502449; doi:10.1186/1471-244X-12-172)
Supplement: Supplementary file 1 [file 1471-244X-12-172-S1.docx]

Appendix 1: Momentary assessment items.

| Set | Construct | Item no (branched questions idented) | |
|---|---|---|---|
| One | Guilt | 1 | I have felt guilty |
| | | 2 | I have been preoccupied with my guilty thoughts |
| | | 3 | I have felt that I deserve punishment' |
| | Hopelessness | 4 | I have felt optimistic about the future (rev) |
| | | 5 | I have felt like there is little point in trying |
| | | 6 | I have felt like the future holds little for me |
| | | 7 | I have felt like giving up |
| | Depression | 8 | I have felt sad' |
| | | 9 | I have felt cheerful (rev) |
| | | 10 | I have felt motivated to do things (rev) |
| | | 11 | My mood has affected my appetite or sleep |
| | | 12 | I have had thoughts about harming myself |
| | Social withdrawal | 13 | I have been socialising with other people |
| | | 14 | I have felt like spending time with others |
| | | 15 | I have approached other people to talk to |
| | Conceptual disorganisation | 16 | People have found it difficult to follow what Im saying |
| | | 17 | My words have been coming out 'jumbled-up' |
| | | 18 | I have found it difficult to keep to a point |
| | Excitement | 19 | I have felt restless. |
| | | 20 | I have found it difficult to stay still' |
| | | 21 | I have found it difficult to concentrate on something |
| | Hallucinations | 22 | I have heard voices |
| | | 23 | I have found it difficult to concentrate on other things |
| | | 24 | This stopped me from doing things |
| | | 25 | Hearing the voice(s) upset me |
| | | 26 | I have seen things that other people can't see |
| | | 27 | I have found it difficult to concentrate on other things |
| | | 28 | This stopped me from doing things |
| | | 29 | Seeing these things upset me |
| | Reactivity | 30 | Keeping the diary has influenced my thoughts |

| | | | |
|---|---|---|---|
| Two | Anxiety | 1 | I have felt worried, nervous or anxious' |
| | | 2 | My heart has been racing or I have been shaking |
| | | 3 | My anxiety has stopped me from doing things |
| | | 4 | This has affected my appetite or sleep |
| | Grandiosity | 5 | Compared to the average person, I am (analogue scale: worse - better). |
| | | 6 | I have felt like I am special |
| | | 7 | I have felt like I have powers or abilities that other people don’t have |
| | Hostility | 8 | I have felt irritable' |
| | | 9 | I have angrily shouted or sworn at someone |
| | | 10 | I have broken something or lashed out |
| | Somatic Concern | 11 | I have been worrying about my physical health |
| | | 12 | I have been sure that something is physically wrong with me |
| | | 13 | I have found it difficult to concentrate on other things |
| | Guilty ideas of reference | 14 | I have felt blamed for something that I didn’t do |
| | | 15 | I have felt held responsible for things that were not my fault |
| | | 16 | I have felt wrongly accused of something |
| | Suspicious thoughts | 17 | I have worried about saying too much |
| | | 18 | I have been suspicious |
| | | 19 | I have felt like someone or something meant me harm |
| | | 20 | This has stopped me from spending time with others |
| | | 21 | This has stopped me from doing things |
| | | 22 | I have found it difficult to concentrate on other things |
| | Delusions* | 23 | [enter primary delusion] |
| | | 24 | This upset me |
| | | 25 | This stopped me from doing things |
| | | 26 | I have found it difficult to concentrate on other things |
| | | 27 | [enter secondary delusion] |
| | | 28 | This upset me |
| | | 29 | This stopped me from doing things |
| | | 30 | I have found it difficult to concentrate on other things |
| | | 31 | Keeping the diary has influenced my mood |

*Delusions selected from the following:

I have felt like I could read other people's thoughts
I have felt like other people were reading my thoughts
I have felt that my thoughts were being controlled or influenced
I have felt like my thoughts were alien to me in some way
I have felt like the world is not real
I have felt like I am not real
I have felt like people were not what they seemed
I have felt like things on the TV, in books or magazines had a special meaning for me
I have felt like there was a conspiracy against me
I have felt jealous
I have felt like something bad was about to happen
